# Tax peptide vaccine suppressed the leukemia in humanized mouse model of ATL

**DOI:** 10.1186/1742-4690-12-S1-O43

**Published:** 2015-08-28

**Authors:** Jun-ichi Fujisawa, Sung-il Lee, Jinchun Yao, Yihua Ren, Masakazu Tanaka

**Affiliations:** 1 Department of Microbiology; 2 Institute of Biomedical Science, Kansai Medical University, Osaka, Japan

## 

Cytotoxic T-lymphocyte (CTL) against HTLV-1 Tax has been demonstrated to play a vital role in controlling HTLV-1 infected cells in the infected carrier people. It was also shown that DNA immunization of mice with Tax expression vector suppressed the tumor growth of EL-4 lymphoma cells expressing Tax (EL4/Gax) through the activation of anti-Tax CTL [1]. Recently we established an HTLV-1-infected humanized mouse model (hu-NOG) to recapitulate ATL development and HTLV-1-specific immunity [2]. Here the efficacy of Tax-peptide vaccination was examined in the hu-NOG mouse model. Mixture of twelve overlapping peptides of 40-42 amino acids long encompassing whole Tax protein was inoculated subcutaneously three times weekly to hu-NOG mouse and then -irradiated HTLV-1 producing Jurkat cells were intraperitoneally injected to infect HTLV-1. While the control mice without immunization died of leukemia within two months of infection, Tax-immunization retarded the out growth of human lymphocytes after HTLV-1 infection and two out offive mice survived with alimited number of infected T-cells. IL-12 induction and the enhanced expression of Tax-specific CD8 T-cell were observed in the peripheral blood of immunized mice before infection. Furthermore, it was shown that the intranasal inoculation of Tax peptides has comparable effect on the onset of leukemia in the hu-NOG systems. These results suggest that the Tax peptide vaccination can elicit protective immunity against HTLV-1 infection and/or ATL developments.

